# Novel Human Parechovirus from Brazil

**DOI:** 10.3201/eid1502.081028

**Published:** 2009-02

**Authors:** Jan Felix Drexler, Klaus Grywna, Andreas Stöcker, Patrícia Silva Almeida, Tereza Cristina Medrado Ribeiro, Monika Eschbach-Bludau, Nadine Petersen, Hugo da Costa Ribeiro, Christian Drosten

**Affiliations:** University Hospital Prof Edgard Santos, Federal University of Bahia, Salvador, Brazil (J.F. Drexler, A. Stöcker); Bernhard Nocht Institute for Tropical Medicine, Hamburg, Germany (J.F. Drexler, N. Petersen); University of Bonn Medical Centre, Bonn, Germany (J.F. Drexler, K. Grywna, M. Eschbach-Bludau, C. Drosten); Federal University of Bahia, Salvador (P. Silva Almeida, T.C. Medrado Ribeiro, H. da Costa Ribeiro, Jr.)

**Keywords:** Picornaviridae, picornavirus infections, human parechovirus, Brazil, genetic diversity, communicable diseases, dispatch

## Abstract

Human parechoviruses (HPeVs) were detected by reverse transcription–PCR in 16.1% of 335 stool samples from children <6 years of age with enteritis in Salvador, Brazil. Whole genome sequencing of 1 sample showed a novel HPeV that has been designated as HPeV8.

The human parechovirus (HPeV) species are small, nonenveloped RNA viruses that belong to the highly diversified family *Picornaviridae* (*1*). HPeV types 1 and 2 had been known as echoviruses 22 and 23 within the genus *Enterovirus* but were recognized in the early 1990s as an independent genus (*2*). Recognition of clinical relevance is increasing after 4 novel types were more recently described (*3**–**7*).

Seroprevalence studies from different countries indicate that almost the entire human adult population is infected. Predominantly in infants, HPeVs can cause a variety of clinical symptoms, including diarrhea, and respiratory infection (*8**,**9*). Recent data point toward substantial involvement in severe conditions, such as meningitis and infant sepsis, for which HPeV may constitute the second most frequent causative virus after enterovirus in young children (*10*). Different HPeV types may cause different clinical diseases (*10**,**11*). Unconnected diseases might be caused by yet unrecognized HPeVs.

## The Study

To identify possibly unrecognized HPeVs, we systematically searched for HPeVs in patients in Brazil with enteritis. We used stool samples for the study because the related enteroviruses are preferentially transmitted through feces. Because reverse transcription–PCR (RT-PCR) for the genus *Enterovirus* cannot detect HPeVs (*9**,**10*), a broad-range real-time RT-PCR assay was developed that can detect all known parechovirus types. The assay also can detect new HPeV types (*9*).

The study cohort comprised 335 stool samples from Brazilian infants and children <6 years of age with acute diarrhea, defined as >3 watery stools in the previous 24 hours and lasting no longer than 13 days. From February 2006 through August 2007, children were seen as outpatients or were hospitalized at the University Hospital in Salvador de Bahia, Brazil, because of severe dehydration. All analyses were performed by using an ABI 7500 real-time RT-PCR (Applied Biosystems, Foster City, CA, USA) and an ABI 3100 automated sequencing platform (Applied Biosystems) at the local Infectious Disease Research Laboratory. Informed consent was obtained from the mothers of all enrolled patients. The study was approved by the institutional ethics committee.

We performed RNA extraction and real-time RT-PCR as described (*9*). A total of 16.1% of samples tested positive for HPeV. Many samples yielded low HPeV RNA concentrations indicated by threshold cycles later than 32 in real-time RT-PCR.

**Figure 1:**
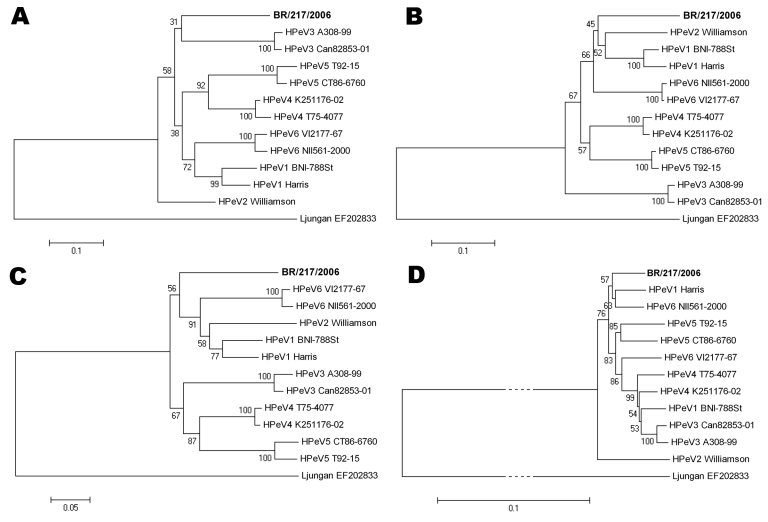
Evolutionary relationships between known parechoviruses and the new human parechovirus from this study (**boldface**). Phylogenetic analyses of A) complete viral protein (VP) 1, B) VP0, and C) VP3, and D) the whole nonstructural region (comprising regions P2 and P3), were constructed using the Jones-Taylor-Thornton matrix-based substitution model on an amino acid–driven alignment (pairwise deletion option). The evolutionary histories were inferred using the neighbor-joining method and are in the units of the number of amino acid substitutions per site. Relevant bootstrap values from 500 replicate trees are shown next to the branches. The scale bars show the evolutionary distance from each root. Analysis was conducted in MEGA software version 4 (www.megasoftware.net). HPeV reference strains are named in full detail; their GenBank accession numbers are not further indicated. Contemporary HPeV1 strain BNI-788st accession number was EF051629 and HPeV6 strain BNI-67 accession number was EU022171. The tree was rooted against Ljungan virus, a rodent parechovirus (GenBank accession no. EF202833). In the P2/P3 tree, branches to the Ljungan virus node have been truncated for space reasons, as indicated by dotted lines.

The viral protein (VP) 1 capsid protein gene has been established for molecular typing of HPeV (*3*). Consensus primers for amplification and sequencing of the VP1 gene were developed. Primer sequences were VP1 forward 5′-CCATARTGYTTRTARAARCCYCT-3′ and VP1 reverse 5′-CARAAYTCDTGGGGYTCMCARATGG-3′. VP1 RT-PCR was successful in only 11 of 54 HPeV-positive samples, consistent with low RNA concentrations in most samples. Ten of the 11 sequenced samples were of known and well-characterized types (type 1, 7 samples; type 5, 2 samples; and type 6, 1 sample). These samples were not further analyzed. One sample showed a VP1 sequence that clustered with none of the known HPeV types in phylogenetic analysis (Figure 1). To determine whether this virus represented a new type, its complete genome except the first 27 nucleotides of the 5′ terminus was amplified by overlapping PCR fragments, and the full nucleotide sequence was determined as described previously (*9*) (GenBank accession no. EU716175).

The best matching sequence was HPeV type 4 with 76.3% aa identity (Table). Lowest aa identity was 69.3% between the new sequence and type 5. Genetic identity to Ljungan virus, a rodent parechovirus, was only 44.5%, comparable to all previously described HPeVs. Phylogenetic segregation from all known HPeVs was obvious not only in VP1 but also along the structural proteins VP0 and VP3 (aa identities with established HPeV types ranges 71.2%–80.2% in VP0 and 74.1%–80.0% in VP3) (Figure 1).

**Table:** VP1 amino acid identity between sequences of HPeV, Brazil*

PeV type and strain	1	2	3	4	5	6	7	8	9	10	11	12	13
HPeV1													
1. Harris													
2. BNI-788St	89.6												
HPeV2													
3. Williamson	78.7	80.0											
HPeV3													
4. A308-99	71.7	71.7	71.7										
5. Can82853-01	72.6	72.1	71.7	97.3									
HPeV4													
6. T75-4077	78.4	74.0	74.8	70.4	71.2								
7. K251176-02	77.9	77.1	76.5	70.4	70.8	96.6							
HPeV5													
8. CT86-6760	74.9	74.0	73.0	66.4	66.8	77.6	80.2						
9. T92-15	74.9	71.4	70.9	65.0	65.5	76.7	77.2	94.8					
HPeV6													
10. NII561-2000	81.4	78.8	73.5	74.8	75.2	73.2	73.2	71.4	71.9				
11. VI2177-67	81.4	77.9	73.5	73.5	73.9	73.2	73.2	72.7	73.2	95.7			
Novel type													
12. BR/217/2006	74.6	75.9	73.7	72.0	72.4	74.1	76.3	72.4	69.3	72.8	72.8		
Rodent PeV													
13. Ljungan	43.5	43.5	48.0	44.4	44.0	42.9	44.6	42.9	42.4	43.1	43.5	44.5	

*HPeV, human parechovirus; PeV, parechovirus; VP1, viral protein 1. Percentage of amino acid identity per site from analysis between sequences is shown. All results (%) are based on the pairwise analysis of 13 sequences (pairwise deletion option). Analyses were conducted in MEGA software (www.megasoftware.net). There were 297 positions in the final dataset.

**Figure 2:**
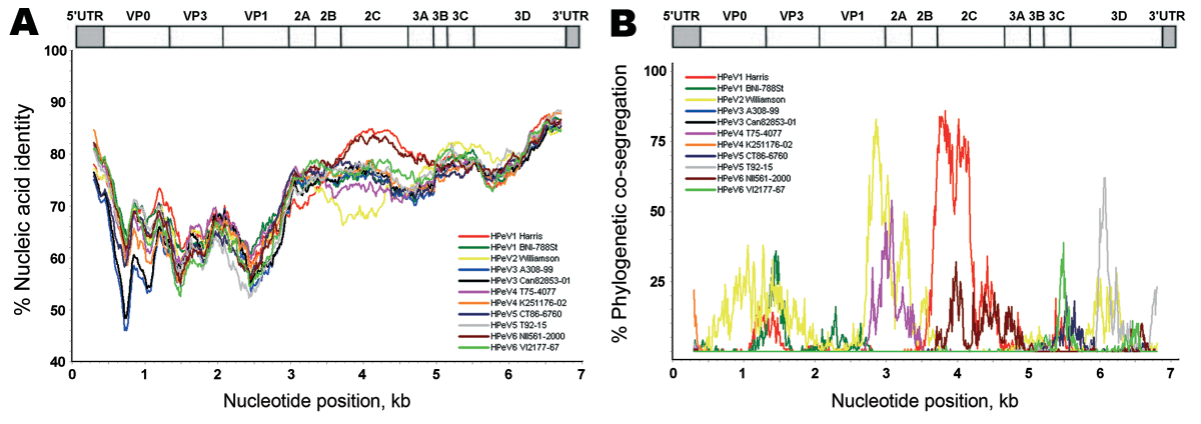
Nucleic acid identity with known parechoviruses. The near full-length genome of the new parechovirus BR/217/2006 was analyzed with SimPlot software (http://sray.med.som.jhmi.edu/SCRoftware/simplot) using a 600-bp sliding window and a step size of 10. Because of partially incomplete 5′ untranslated (UTR) region GenBank reference sequences, an approximate 400 nucleotides had to be cut from the 5′ end of all genomes. HPeV, human parechovirus. A) Nucleic acid identity, per analysis window, for strain BR/217/2006 with prototype strains. Nucleotide positions on x-axis show the center of the window. B) BootScan analysis using the same window settings. A bootstrapped phylogenetic analysis was conducted per window along the alignment. Graphs represent the percentage (bootstrap values) at which each strain cosegregates phylogenetically in the analysis window with strain BR/217/2006. Prototype strains used for comparison are shown in the inserts in each panel. A schematic representation of the parechovirus genome is given on top.

Nonstructural family *Picornaviridae* genes are highly recombined (*1**,**3**,**12**,**13*), resulting in a mosaic structure that limits their utility in phylogenetic analysis. This characteristic also was the case for the whole nonstructural region (comprising regions P2 and P3) of the novel virus. It was not clearly segregated from that of all other HPeV prototype strains, showing closest overall relationship with HPeV type 1 strain Harris and the HPeV6 prototype strain NII561-2000 (Figure 1). To identify possible recombination in the P2/3 region of the novel virus, we conducted Sim Plot analysis (http://sray.med.som.jhmi.edu/SCRoftware/simplot) (Figure 2). We found no clear similarity with any of the established HPeV prototype strains. In contrast to other HPeV prototype strains, BootScan analysis showed no evidence for recombination with other prototype strains in the nonstructural gene region. However, this analysis could also not exclude recombination with any other HPeV because of the small number of HPeV full genome sequences currently available.

The new HPeV lacked a typical RGD (Arg-Gly-Asp) aa motif in the VP1 C terminus. This motif has proven important for HPeV type 1 infectivity, presumably because of interaction with cellular receptors (*14*). Such a motif is present in all known HPeV strains except type 3, and some researchers have suggested that the latter may use a different receptor for cell entry (*3**,**13*).

## Conclusions

Most HPeV types have been identified only recently. The associated spectrum of diseases is not fully understood and probably has been underestimated. Recent data indicate that HPeVs may cause severe clinical conditions, such as infant sepsis and meningitis, in addition to acute diarrhea (*10*). Prevalence in young children with diarrhea was >16% in previous studies; more important, <8% of meningitis cases showed evidence of HPeV (*9**–**11*). The molecular ecology of HPeV seems especially relevant in view of their diversified and strain-dependent pathogenesis (*10**,**11*).

This report on HPeVs from Brazil confirms their global distribution. The level of diversification between the novel parechovirus and established HPeV types is clearly higher than the 20% aa distance in the VP1 protein, which resembles the distance between serotypes of enteroviruses (*1**,**3*) and exceeds the definition threshold of HPeV types (*3*). During revision of this report, the virus received the designation HPeV8 by the ICTV Picornavirus Study Group (www.picornastudygroup.com/types/parechovirus/hpev.htm). Like HPeV3, HPeV8 lacks the RGD motif; some researchers have suggested that HPeV3 may use a different receptor than other HPeV types for cell entry (*3**,**13*). Of all HPeV types, type 3 has been most strongly associated with severe neurologic and systemic clinical conditions (*10**,**11**,**15*). The lack of an RGD motif might implicate a different cell or tissue tropism for HPeV8 as well. The search for unknown HPeVs should be extended to other clinical conditions thus far not associated with HPeV.
